# Metastatic colorectal cancer and type 2 diabetes: prognostic and genetic interactions

**DOI:** 10.1002/1878-0261.13122

**Published:** 2021-11-19

**Authors:** Alessandro Ottaiano, Luisa Circelli, Mariachiara Santorsola, Giovanni Savarese, Daniela Fontanella, Valerio Gigantino, Annabella Di Mauro, Maurizio Capuozzo, Silvia Zappavigna, Angela Lombardi, Francesco Perri, Marco Cascella, Vincenza Granata, Maurizio Capuozzo, Guglielmo Nasti, Michele Caraglia

**Affiliations:** ^1^ Istituto Nazionale Tumori di Napoli, IRCCS “G. Pascale,” Naples Italy; ^2^ AMES, Centro Polidiagnostico Strumentale srl Naples Italy; ^3^ Molecular Biology Innovalab Scarl Naples Italy; ^4^ Department of Precision Medicine University “L. Vanvitelli” of Naples Italy; ^5^ Cytometric and Mutational Diagnostics Azienda Universitaria Policlinico “L. Vanvitelli,” Naples Italy; ^6^ Department of Pharmacy ASL‐Naples‐3 Ercolano Italy; ^7^ Laboratory of Precision and Molecular Oncology Biogem Scarl Institute of Genetic Research Ariano Irpino Italy

**Keywords:** genes, metastatic colorectal cancer, oligo‐metastatic colorectal cancer, prognosis, survival, type 2 diabetes

## Abstract

The present study was undertaken to analyze prognostic and genetic interactions between type 2 diabetes and metastatic colorectal cancer. Patients’ survival was depicted through the Kaplan–Meier product limit method. Prognostic factors were examined through the Cox proportional‐hazards regression model, and associations between diabetes and clinical‐pathologic variables were evaluated by the χ^2^ test. In total, 203 metastatic colorectal cancer patients were enrolled. Lymph nodes (*P = *0.0004) and distant organs (> 2 distant sites, *P* = 0.0451) were more frequently involved in diabetic patients compared with those without diabetes. Diabetes had an independent statistically significant negative prognostic value for survival. Highly selected patients with cancer and/or diabetes as their only illness(es) were divided into three groups: (a) seven oligo‐metastatic patients without diabetes, (b) 10 poly‐metastatic patients without diabetes, and (c) 12 poly‐metastatic diabetic patients. These groups of patients were genetically characterized through the Illumina NovaSeq 6000 (San Diego, CA, USA) platform and TruSigt™Oncology 500 kit, focusing on genes involved in diabetes and colorectal cancer. Gene variants associated with diabetes and cancer were more frequent in patients in group 3. We found that type 2 diabetes is a negative prognostic factor for survival in colorectal cancer. Diabetes‐associated gene variants could concur with malignancy, providing a rational basis for innovative models of tumor progression and therapy.

## Graphical abstract

Type 2 diabetes (T2D) is a frequent comorbidity among metastatic colorectal cancer patients. Here we show that T2D is a negative prognostic factor and hypothesize that genetic polymorphisms involved in diabetes could concur with malignancy providing new insights for innovative models of tumor progression and therapy in this subset of patients.

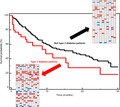

**Abbreviations**
ACMG/AMP: American College of Medical Genetics and Genomics and the Association for Molecular Pathology
CEA: carcino‐embryonic antigen
CI: confidence intervals
CR: complete response
CRC: colorectal cancer
DC: disease control
DNA: deoxyribonucleic acid
ECOG: Eastern Cooperative Oncology Group
ESMO: European Society of Medical Oncology
HLA: histocompatibility leukocytes antigens
HR: hazard ratio
IGFs: insulin‐like growth factors
mCRC: metastatic colorectal cancer
MODY: maturity onset diabetes of the young
NGS: next generation sequencing
omCRC: oligo‐metastatic colorectal cancer
OS: overall survival
PD: progressive disease
PFS: progression‐free survival
PR: partial response
PS: performance status
Rs: reference SNP cluster id
SD: stable disease
T1D: type 1 diabetes
T2D: type 2 diabetes
tbCT: total body computed tomography

## 1 Introduction

Diabetes is a diffuse disease in Western countries. In Europe, about 10% of adults live with diabetes: the highest prevalence is recorded in Germany (14.2%) while the lowest in Ireland (4.4%). A prevalence of about 14% in adults is documented in the USA. These data account for more than 100 million people affected by diabetes in Western countries. About 90% have type 2 diabetes (T2D), the remaining type 1 [1]. Well‐known risk factors for developing T2D are low socioeconomic status, older age (> 65 years), male sex, and obesity. On the other hand, type 1 diabetes (T1D) is equally distributed between different genders and its prevalence increases north of the equator, as the lower the temperature is and the longer the cold seasons last. Hyperglycemia is the most common sign of diabetes influencing micro‐ and macro‐vascular generalized damages with subsequent acute (ketoacidosis and hyperosmolar syndrome) and chronic complications (nephropathy, neuropathy, vasculopathy, etc.) [2]. However, the etiopathogenesis of diabetes is heterogeneous. T2D is a complex poly‐genic disease resulting from multiple defects or variants (largely with small effect size) in pathways involved in glucose metabolism regulation interacting with lifestyle and environmental exposures [3]. T1D is an autoimmune disease against pancreatic beta‐islets [4]. Involved genes include HLA (histocompatibility leukocyte antigens) and regulators of tolerance and immune responses (*IL‐2R, CCR7, IL‐10, HORMAD2,* etc.). On the other, maturity onset diabetes of the young (MODY) is a monogenic disease where pathogenic mutations in specific genes (*GCK, HNF1A, HNF4A, etc*.) have been identified [5]. In this case, the genetic assessment can guide both diagnosis and appropriate therapy. Diabetes is one of the features of the so‐called “Metabolic Syndrome” that is a cluster of metabolic and hormonal factors having a central role in the initiation and recurrence of many Western chronic diseases, including cancer, and is considered the world's leading health problem in the coming years [6]. Moreover, it was shown that metabolic syndrome is correlated with a higher risk of colorectal adenoma, especially in old (≥ 50 years) male patients, potentially benefiting from behavioral interventions in preventing the development of colorectal cancer (CRC) [7]. Interestingly, previous studies evidenced a correlation among T2D and the occurrence and clinical outcome of CRC [8, 9, 10].

The latter is the third most common cancer in Western countries, as in 2018 about 1.8 million people received a diagnosis of CRC and 881 000 died. The male/female ratio is 1.42, with an incidence peak at 65 years [11]. Interestingly, in the last decade, in low/middle‐income countries, the incidence is increasing, while in high‐income ones both the incidence and mortality are decreasing because of risk‐factor reduction (particularly smoking and red meat consumption) and amelioration through screening tests and treatments. Notably, it is estimated that about half of CRCs could be attributable (and consequently avoidable) by lifestyle. Well‐known risk factors are vitamin D deficiency, diet high in animal fat and low in vegetables and fruits, smoking, diabetes, obesity, and *Helicobacter pylori* infection [12, 13, 14]. The etiopathogenesis of CRC resides in the accumulation of gene mutations prompting progressive genetic trajectories from normal mucosa to adenocarcinoma, with a fully malignant phenotype able to produce distant metastases (“Vogelstein model”) [15].

Most T2D patients develop beta‐cells insulin impaired secretion and/or resistance associated with an IGFs (insulin‐like growth factors) serum compensatory release. In epidemiologic and pragmatic point of views, about 20% of CRC patients have T2D and, conversely, diabetes is a well‐known risk factor for CRC development and progression [16].

The present study was undertaken to analyze the prognostic role of T2D in advanced CRC and to generate hypotheses on the genetic links existing between these two diseases through a regulated and rigorous genotype/phenotype correlation.

## 2 Materials and methods

### 2.1 Patient management and selection

This study was officially approved by the Scientific Directorate on November 11, 2020 (TIMA 25/20). The prognostic role of T2D in a selected series of 203 patients was analyzed. Patients were managed at the SSD (*Struttura Semplice Dipartimentale*) of Innovative Therapies for Abdominal Metastases of the *Istituto Nazionale Tumori di Napoli, IRCCS “G. Pascale”* and at the University of Campania *“L. Vanivitelli”* according to the ESMO (European Society of Medical Oncology) guidelines [17]. Oligo‐metastases were defined as one to three lesions per organ with a maximum tumor diameter smaller than 70 mm and no single lesions more than 25 mm diameter; otherwise, the disease was defined as poly‐metastatic [18, 19]. Patients with Performance Status (PS) ECOG (Eastern Cooperative Oncology Group) ≥ 2, age > 80 years, and life expectancy < 3 months were excluded to avoid negative prognostic interferences. To avoid unexpected and unpredictable prognostic influences, patients bearing *BRAF* mutated tumors were not included in this cohort. The study was conducted according to the standards set by the Declaration of Helsinki. All patients gave written informed consent before treatments and genetic assessments.

### 2.2 Patients follow‐up

All patients were monitored with total body computed tomography (tbCT) scan and CEA (Carcino‐Embryonic Antigen) every 3 months. RECIST (Response Evaluation Criteria In Solid Tumors v. 1.1) [20] were applied to classify radiologic responses. Complete response (CR) was defined as complete remission of all lesions on tbCT. Partial response (PR) was defined as at least a 30% reduction in the sum of target lesions diameters. Progressive disease (PD) was defined as an increase in the sum of target lesions diameters of at least 20%. All changes between 30% decrease and 20% growth were defined as stable disease (SD). Disease control (DC) was the sum of CR+PR+SD.

### 2.3 Tumor specimens, NGS (next‐generation sequencing), and genotype/phenotype correlation

Formalin‐fixed and paraffin‐embedded (FFPE) tissue specimens of metastases (biopsies or resected lesions) from CRC were collected from selected patients. Three groups were identified: (a) oligo‐metastatic patients having cancer has the only illness, (b) poly‐metastatic patients having cancer and T2D as the only illnesses, (c) poly‐metastatic non‐T2D patients having cancer as the only illness. Thus, patients with hypertension and/or other cardiovascular diseases, chronic infections, autoimmune, or inflammatory diseases, other cancers, were excluded from genetic characterization. Informed consent to perform genetic assessments was obtained from all patients. Microdissection of tumor cells in serial sections of 10 µm was performed under a morphological control for each tissue specimen. The MGF03‐Genomic DNA FFPE One‐Step Kit, according to the manufacturer’s protocol (MagCoreDiatech, Diatech Lab Line, Jesi, Italy), was applied to isolate DNA; its quality was assessed using the FFPE QC Kit (Illumina, San Diego, CA, USA). Libraries were constructed through the TruSigt™Oncology 500 kit that is a robust and comprehensive genomic profiling performed in NGS and targeting 523 cancer‐relevant genes (the complete list is reported in Fig. S1). The study focused on genes shared by T2D and cancer: *CCND2, CDKN1B, CDKN2A, CDKN2B, CENPA, EML4, ID3, HNF1A, IGF1, IGF2, IGF1R, INSR, IRS1, IRS2,* and *TCF7L2* (Table S1). The assay is able to detect indels, small nucleotide variants (SNVs), splice variants, copy‐number/structural variations, and gene fusions. An Illumina NovaSeq 6000 platform was used to perform sequencing.

### 2.4 Diabetes definition

Patients classified as T2D were all over 40 years, did not require insulin administration, and were initially diagnosed according to the American Diabetes Association criteria (casual plasma glucose concentration ≥ 200 mg·dL^−1^ or fasting plasma glucose ≥ 126 mg·dL^−1^ or 2‐h glucose ≥ 200 mg·dL^−1^ after the Oral Glucose Tolerance Test) [21, 22].

### 2.5 Statistical analyses, study design, and data presentation

Overall survival (OS) was the primary outcome and was assessed from the diagnosis of advanced disease until death from CRC (cancer‐specific survival). Progression‐free survival (PFS) was not considered as a study objective for the following reasons: (a) the vital status is a more solid and reliable outcome to analyze, (b) treatments and radiologic evaluations were heterogeneous. Data were extracted from an internal electronic database reporting clinical records of metastatic CRC patients. The enrolment of patients was limited to the last 5 years to avoid any prognostic influences of therapeutic, diagnostic, and methodological changes occurring physiologically over time in clinical practice. With a test power of 80%, an alpha value of the I‐type error of 5%, a 1‐year survival of 80% (in unselected mCRC patients), a hazard ratio (HR) of 0.62 between the two clinical settings (T2D *vs* non‐T2D patients), the minimum required sample size was 36 in the T2D arm and 160 in the non‐T2D arm. The statistics applied for a sample size calculation was a chi‐square‐based algorithm for proportions comparison available in medcalc ^®^ 9.3.7.0 software (MedCalc Software Ltd, Ostend, Belgium). Subsequently, a two‐tailed log‐rank test with an alpha value of the I‐type error of 5% was applied to verify statistical significance at univariate analysis. OS was depicted through the Kaplan–Meier product limit method. Dichotomized prognostic factors (covariates) were the following: age (< 65 *vs* ≥ 65), gender (male *vs* female), response to first‐line chemotherapy (disease control *vs* no diseases control), *RAS* mutations (mutated *vs* non‐mutated), side (left *vs* right), metastatic involvement (1 site *vs* > 1). Their prognostic interactions on OS were examined through multivariable analyses based on the Cox proportional‐hazards regression model. The estimate of the survival probability according to covariates were expressed through the HRs intended as the risk of death, at any time, for a patient with the risk factor present compared to a patient with the risk factor absent (given both patients identical for all other covariates). HRs were reported in univariate and multivariable analyses with 95% confidence intervals (CIs). Statistical analyses and Kaplan–Meier curves were performed using the medcalc ^®^ 9.3.7.0 and Excel software. Associations between T2D and clinical and pathologic variables were evaluated by the χ^2^ test. *P* < 0.05 was considered statistically significant.

### 2.6 Bioinformatics analysis

The Illumina TruSight Oncology 500 bioinformatics pipeline was used to analyze and interpret sequencing results. The coverage in the target region and the generated reads were above the manufacturer’s threshold (150× and > 100 millions, respectively). The human reference genome to align sequences was GRCh37 (http://www.ncbi.nlm.nih.gov/projects/genome/assembly/grc/human/index.shtml) by applying the Burrows‐Wheeler Aligner tool with default parameters [23]. Genetic variants were intersected with GENCODE, ICGC‐PCAWG, dbNSFP, COSMIC, 1000Genomes, CancerMine, ClinVar, OncoScore, CIViC, and CBMDB databases to assess their clinical significance. Variants were removed in case of global minor allele frequency < 1% after filtering with unmatched normal datasets. Variants prioritization was done according to the four‐tiered structure of the ACMG/AMP [24]. The study was focused on variants of *CCND2, CDKN1B, CDKN2A, CDKN2B, CENPA, EML4, ID3, HNF1A, IGF1, IGF2, IGF1R, INSR, IRS1, IRS2,* and *TCF7L2* genes (see above). No known “pathologic” variants were found according to ACMG/AMP prioritization (strong or potential clinical significance, Tier 1 or 2); however, variants were also manually analyzed to exclude false negatives. The reported variants are “benign” or “unknown.”

Variants were reported with the consequent protein change. In specific cases (*i.e*. 5′ UTR or intron variant), variants were reported with the genomic change (c.). The Phenolyzer tool was used to reveal relationships between any “seed” genetic variants and “secondary” ones. Phenolyzer adds insight into prioritization and interpretation of genetic variants. It interrogates and connects some crucial gene‐disease databases (OMIM, Orphanet, ClinVar, Gene Reviews, and GWAS Catalog) and prioritizes genes on the basis of updated scientific knowledge (sharing of biological pathways or gene family, gene–gene transcriptional regulation, protein–protein interactions, etc.). Results are expressed through a score system (see the end of each bar in the specific graph) and a network visualization picture that provides readers with an intuitive panoramic view of the weighted interactional context (the legend is reported in Fig. S2). The https://phenolyzer.wglab.org/ open access site was accessed on June 1, 2021. The following parameters were used to study genes interaction: disease/phenotype, colorectal cancer; seed genes interaction, DisGeNET database and GAD (Genetic Association Database); gene scores interaction, GHS (Gene Haploinsufficiency Score), and GIS (Gene Intolerance Score). However, for a complete methodology description of this computational tool, see Yang *et al*. [25].

## 3 Results

### 3.1 Clinicopathologic characteristics of the selected series

In all, 203 mCRC patients were studied and 41 were affected by T2D. Clinicopathological characteristics of patients and tumors according to the presence of T2D are shown in Table 1. More than half of the patients were older than 65 years (53.2%). Genders were quite equally distributed (48.2% male; 51.8% female). Although not significant, there was a prevalence of obesity in T2D patients (17.0% *vs* 6.7% in not diabetic patients). G3 grading tumors were predominant (85.7%) and 64% of tumors derived from the right colon. The largest part of our series had pT3 tumors (40.8%) at initial pathologic staging. Sixty‐three percent of patients had metastases to locoregional lymph nodes. There was a significant association between lymph nodes involvement and T2D (*P* = 0.0004). *RAS* status assessed on primary resected tumors or biopsies from metastases was available in all cases. Most tumors were *KRAS* mutant and *NRAS* wildtype (56.6%) without differences between T2D and not T2D.

**Table 1:** Clinicopathological characteristics according to the presence or not of T2D. pT, pathological staging of primary tumor according to AJCC; F, female; LN, lymphnodes; M, male; mut, mutant; MS, microsatellite; wt: wildtype.

Characteristic	T2D		*P* ^a^
	No	Yes	
Age			
< 65	75	20	
≥ 65	87	21	0.7764
Gender			
M	73	25	
F	89	16	0.0692
Body‐mass index			
Normal (18.5–24.9)	68	12	
Overweight (25–29.9)	83	22	
Obese (≥ 30)	11	7	0.0714
Grading			
G1/G2	21	8	
G3	141	33	0.2856
Side of primary tumor			
Left	59	14	
Right	103	27	0.7869
pT^b^			
pT1/pT2	30	7	
pT3	68	15	
pT4	47	18	0.3345
LN involvement^b^			
0	50	3	
1–3	62	14	
> 3	33	19	0.0004
RAS status			
KRAS mut‐NRAS wt	90	25	
KRAS wt‐NRAS wt	69	14	0.3963
KRAS wt‐NRAS mut	2	1	
KRAS mut‐NRAS mut	1	1	
MS status			
Stable	146	37	
Unstable	5	1	
Unknown	11	3	0.9706

^a^ *P* at chi‐square test.

^b^ The row sum does not correspond to the total number of patients because some of them (22) did not receive surgical removal of primary tumor.

### 3.2 Relationships between T2D, tumor burden, and response to therapy

Since both initial metastatic involvement and response to first‐line therapy are clinical factors with crucial prognostic information, their association to T2D was explored. Interestingly, T2D patients were more susceptible to develop high metastatic involvement (> 2 sites, *P* = 0.0451). Similarly, oligo‐metastatic disease, although not significant (*P* = 0.0762), was more frequent in not T2D (15.4%) compared to T2D patients (4.8%). The most applied first‐line chemotherapy was based on chemotherapy (fluoropyrimidines, oxaliplatin, and/or irinotecan) plus bevacizumab (53.2%). T2D was associated with poor response to first‐line chemotherapy (*P* = 0.0103) and a smaller cumulative number of therapy lines (*P* < 0.0001) (Table 2).

**Table 2:** Tumor burden and response to first‐line chemotherapy according to the presence or not of T2D. CR, complete response; CT, chemotherapy; EGFR, epidermal growth factors; NA, not assessable.

Characteristic	T2D		*P* ^a^
	No	Yes	
Metastatic involvement			
One site	32	6	
Two sites	62	9	
> 2 sites	68	26	0.0451
Oligo‐metastatic disease			
Yes	25	2	
No	137	39	0.0762
Type of first‐line CT			
CT	18	8	
CT + anti‐EGFR drugs	54	12	
CT + bevacizumab	88	20	0.3380
Best response to first‐line CT			
CR, PR or SD	92	15	
PD	55	23	0.0103
NA	15	3	
No. of chemotherapy lines			
One	16	17	
Two	17	13	
> 2	127	10	< 0.0001

^a^ *P* at chi‐square test. The row sum does not correspond to the total number of patients because three did not receive chemotherapy (for personal reasons).

### 3.3 Toxicity and treatment exposure

Since the prognostic power of T2D could be due to hypothetic higher toxicity and/or lower doses of chemotherapy compared to not diabetic patients, we analyzed the side effects and treatment exposure in the studied cohort (Tables S2 and S3). Interestingly, no statistically significant differences were found. Neutropenia, asthenia, and diarrhea were the most common G3/G4 adverse events (21 in not T2D, 6 in T2D patients) in first‐ and second‐line therapies. A capecitabine‐induced coronary artery vasospasm resolved with a 25% dose reduction was registered in a T2D patient. Overall, chemotherapy was reduced in 55 patients (42 not T2D and in 13 T2D patients); in both clinical settings, most of the dose reductions (32/42 in not T2D and in 9/13 T2D patients) were caused by hematological toxicity ≥ G2. Chemotherapy delays lasting more than 2 weeks occurred in 29 not T2D and 10 T2D patients.

### 3.4 Prognostic impact of T2D in mCRC patients

**Fig. 1:**
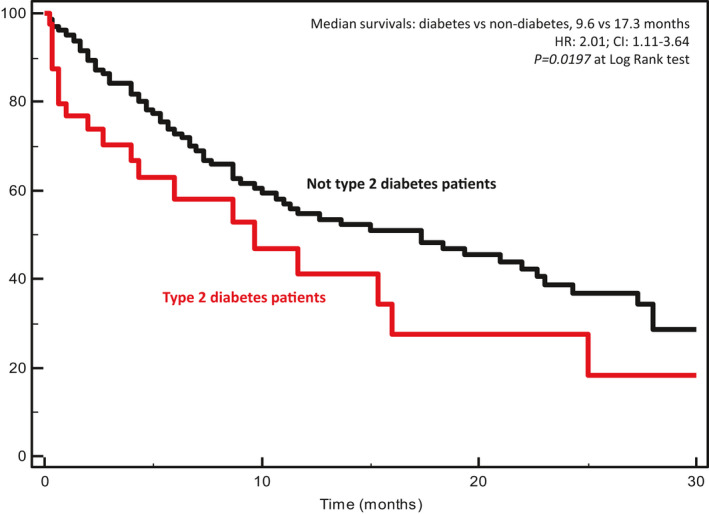
Kaplan–Meyer survival curves according to the presence of type 2 diabetes in metastatic colorectal cancer patients. Median survivals, hazard ratios (HR), confidence intervals (CI), and *P* at log‐rank test are embedded in the figure.

The prognostic role of T2D was explored in 203 mCRC patients with PS ECOG 0–1, life expectancy > 3 months and age < 80 years. After a median follow‐up of 28 months, there were 90 deaths. Kaplan–Meyer curves for OS showed a clear detrimental effect of T2D (Fig. 1). Median survivals for OS (mOS) were 9.6 and 17.3 months for T2D and not T2D patients, respectively (*P* = 0.0197 at log‐rank test; HR: 2.01; CI: 1.11–3.64). At multivariate analysis, adjusted for age (< 65 years *vs* ≥ 65 years), gender (male *vs* female), side of primary tumor (left *vs* right), metastatic involvement (one site *vs* multiple sites), and response to first‐line chemotherapy (disease control *vs* no disease control), T2D (T2D *vs* not T2D) maintained an independent prognostic value (*P* = 0.0226). HRs with CIs for each covariate are shown in Table 3.

**Table 3:** Univariate and multivariable analyses of T2D prognostic impact. F, female; L, left; M, male; R, right.

Co‐variate	Dichotomization	Median survivals	No. of events/patients	*P* at univariate	HR	95% CI	*P* at multivariate
Age	< 65 years *vs* ≥ 65 years	15.3 *vs* 12.6	42/95 *vs* 48/108	0.3141	1.22	0.75–1.99	0.4098
Gender	M *vs* F	13.6 *vs* 15.0	45/98 *vs* 45/105	0.7870	0.82	0.51–1.32	0.4276
Side	L *vs* R	15.3 *vs* 12.6	31/73 *vs* 59/130	0.3431	1.33	0.80–2.19	0.2584
Metastatic involvement	1 site *vs* > 1	27.3 *vs* 9.0	15/38 *vs* 75/165	0.0002	2.36	1.30–4.27	0.0043
Response to firs‐line CT	DC *vs* not DC	22.6 *vs* 8.6	43/107 *vs* 34/78	0.0005	0.50	0.30–0.82	0.0065
Glucose metabolism status	T2D *vs* not T2D	9.6 *vs* 17.3	21/41 *vs* 69/162	0.0197	2.01	1.11–3.64	0.0226

### 3.5 Genetic characterization of poly‐metastatic patients

We tested genetic variants of genes involved in T2D (*CCND2, CDKN1B, CDKN2A, CDKN2B, CENPA, EML4, ID3, HNF1A, IGF1, IGF2, IGF1R, INSR, IRS1, IRS2, TCF7L2*) in 12 pmCRC patients with T2D and 10 pmCRC patients without diabetes (both had cancer as exclusive comorbidity) to address their involvement in CRC progression (see Materials and methods for patient selection). In addition, a very clean clinical model of seven omCRC patients having only one metastatic lesion (liver or lung) and, similar to the previous 22 characterized patients, cancer as the only illness, was characterized.

**Fig. 2:**
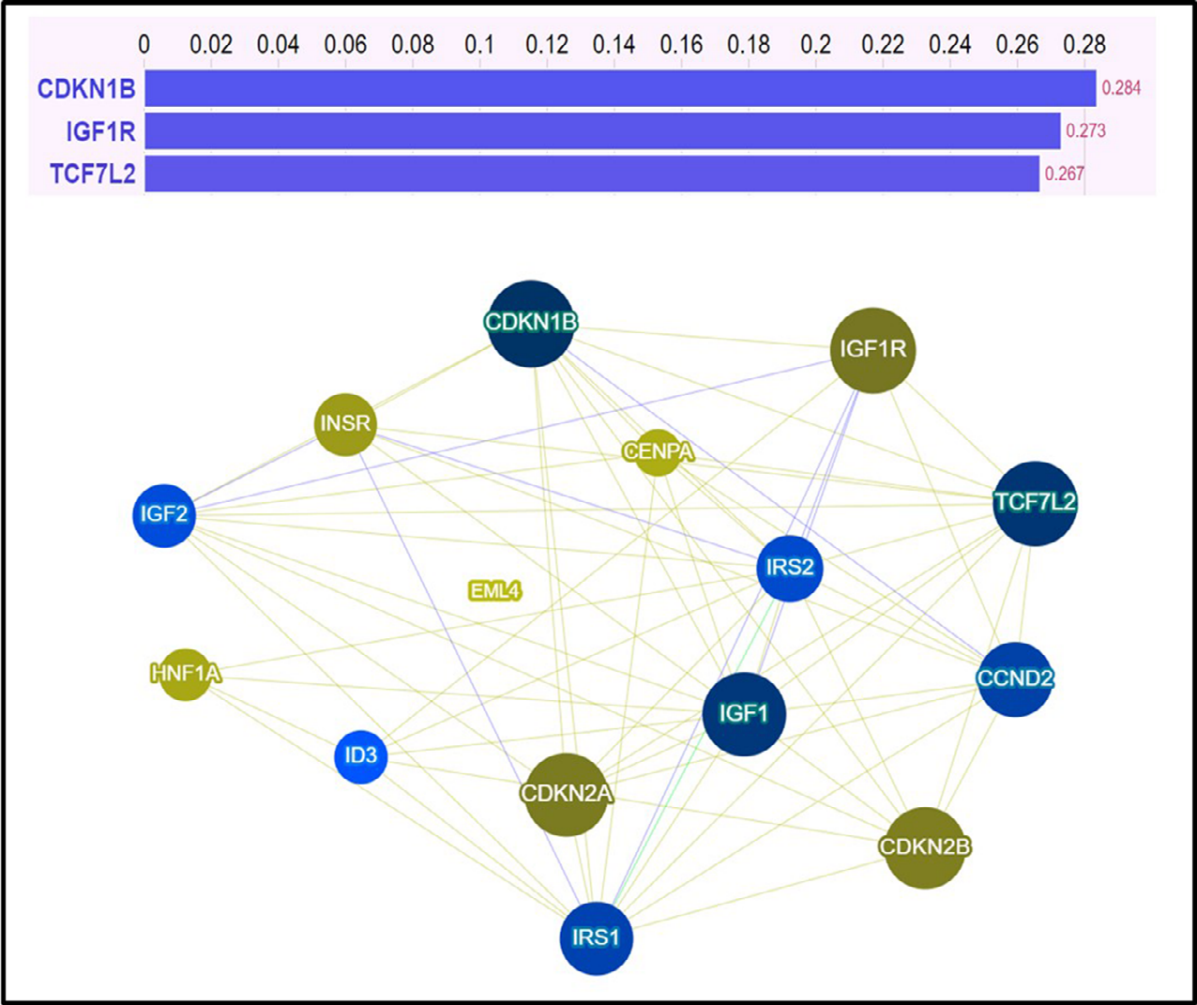
Prioritization of studied genetic variants according to the Phenolyzer tool. In the upper section the reported Phenolyzer score ranged from 0 to 1 (the greater the score, the stronger the association with colorectal cancer). In the lower panel is reported a network visualization tool depicting gene–gene and gene–disease relationships (see Materials and methods).

Genetic results of benign or unknown genetic variants (according to the four‐tiered structure of the AMP/ACMG) of the above‐cited genes included in the TrusightOncology™ 500 panel are reported in Table 4. The analysis is descriptive and hypothesis‐generating. A score representing the sum of the variants revealed in each patient was built. The arithmetic means of this cumulative score in (a) om, (b) pm not T2D, and (c) pm T2D patients were, respectively: 3.1, 5.1, and 11.0. According to Phenolyzer tool, the most important and interrelated genes were: *CDKN1B, IGF1R,* and *TCF7L2* (Fig. 2). Interestingly, among these high‐priority genes, *TCF7L2* variants were never present in omCRC patients.

**Table 4:** Genetic characterization of *CCND2, CDKN1B, CDKN2A, CDKN2B, CENPA, EML4. ID3, HNF1A, IGF1, IGF2, IGF1R, INSR, IRS1, IRS2, TCF7L2* in oligo‐ and poly‐metastatic CRC patients. B, benign; LB, likely benign; NA, not applicable; NR, not reported; RF, risk factor; Pt, patient; vsg, variant stop gained.

	ClinVar Interpretation	Oligo‐metastatic not T2D patients							Poly‐metastatic not T2D patients										Poly‐metastatic T2D patients											
		Pt 1	Pt 2	Pt 3	Pt 4	Pt 5	Pt 6	Pt 7	Pt 8	Pt 9	Pt 10	Pt 11	Pt 12	Pt 13	Pt 14	Pt 15	Pt 16	Pt 17	Pt 18	Pt 19	Pt 20	Pt 21	Pt 22	Pt 23	Pt 24	Pt 25	Pt 26	Pt 27	Pt 28	Pt 29
CCND2																														
c.570C>G (rs3217805)	B																													
CDKN1B																														
p.V109G (rs2066827)	B																													
CDKN2A																														
c.‐191G>A (5′UTR)	NA																													
CDKN2B																														
p.P71L (rs not found)	NR																													
CENPA																														
No variants found	NA																													
EML4																														
p.I382V (rs10202624)	NR																													
p.Q222E (rs not found)	NR																													
p.K283Q (rs6736913, variant 1)	NR																													
p.K283E (rs6736913, variant 2)	NR																													
p.K398R (rs28651764)	NR																													
p.K409R (rs not found)	NR																													
c.1941A>G (3′UTR)	NA																													
c.1909A>C (3′UTR)	NA																													
c.1129C>T (3′UTR)	NA																													
c.‐30A>G (5′UTR)	NA																													
ID3																														
p.T105A (rs11574)	NR																													
HNF1A																														
p.I27L (rs1169288)	B																													
p.S487N (rs2464196)	B																													
p.S574G (rs1169305, variant 1)	B																													
p.S605G (rs1169305, variant 2)	B																													
c.51C>G (rs1169289)	B																													
c.1375C>T (rs2259820)	B																													
IGF1																														
c.102417845G>T (rs6213)	LB																													
IGF2																														
c. 2135427C>G (rs1590118919)	LB																													
IGF1R																														
p.V982I (rs not found)	NR																													
c.2700C>T (rs56400113)	B																													
c.1323G>A (rs not found)	NR																													
c.1686G>A (rs2228531)	B																													
c.903C>A (rs2229764)	B																													
c.2298C>T (rs3743262)	B																													
c.3129G>A (rs2229765)	B																													
INSR																														
p.A2G (rs7508518)	B																													
c.3255C>T (rs1799817)	B																													
c.3033C>T (rs1799815)	B																													
c.2007C>T (rs2962)	B																													
IRS1																														
p.G971R (rs1801278)	RF																													
c,2679G>C (rs35909627)	NR																													
c.2412A>G (rs1801123)	NR																													
IRS2																														
p.G1057D (rs1805097)	RF																													
c.2448T>C (rs not found)	NR																													
c.2487C>T (rs12853546)	NR																													
c.2169C>T (rs not found)	NR																													
TCF7L2																														
p.P460T (rs not found)	NR																													
c.348C>T (rs143305771)	B																													
p.Q321vsg (rs773954064)	NR																													
Score		5	5	2	3	3	2	2	5	5	6	6	4	4	5	4	7	5	14	11	11	13	9	10	10	14	8	12	9	11

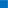
: Phenolyzer high priority genes; 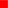: variant present; 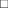: variant not present.

The grey shade indicates that no variants have been found for this gene.

## 4 Discussion

We previously reported that T2D patients were more prone to experience CRC distant recurrence after capecitabine and oxaliplatin‐based adjuvant chemotherapy [26]. In the present study, we focused on metastatic CRC and T2D exploring putative prognostic interactions and genetic connections. A recent meta‐analysis including more than 80 studies showed a significant increase in cancer‐specific mortality (HR, 1.11; 95% CI: 1.05–1.17) and relapse (HR, 1.09; 95% CI, 1.02–1.16) of patients with CRC and diabetes. However, in the cited and analyzed studies stages of disease and type of diabetes were heterogeneous, including all stages and type I/MODY diabetes, respectively [27]. Links between CRC and T2D are largely studied and discussed in the literature [28, 29, 30, 31, 32, 33]. However, the basis of such links still remains largely unknown.

We hypothesized that several genes involved in T2D could have a role in the malignant phenotype, but this is a still fascinating and under investigation concern. Genotype/phenotype correlation studies may help to shed light on this issue. However, these kind of studies are very challenging due to both extreme clinical heterogeneity of the CRC patients and genetic interferences of other comorbidities. A way to overcome these methodological difficulties is to select “clean” clinical models having cancer has the only illness. The characterization of a not diabetic omCRC clinical model in our study allowed us to build a genetic comparator reinforcing the methodology to explore our hypothesis. However, this selection is complicated, and makes very difficult the enrolment of patients into genetic studies. In fact, considering all patients submitted to lung or hepatic surgery in our institute for mCRC from 2013 to 2018, less than 5% had oligo‐metastases with CRC as the only illness during their life. Furthermore, in our clinical setting only 22/203 (10.8%) patients had cancer and/or T2D as the only illnesses. The observation of genetic results of the CRC oligo‐metastatic setting suggests that the selected genetic variants are less present compared to the poly‐metastatic disease. This observation is intriguing, as it adds further complexity to the phenotype of cancer transformation processes. In fact, previous genome‐wide association studies suggested that several “benign” genetic variants in different loci can influence diabetes through a cumulative effect on beta cell function, glucose metabolism, adipogenesis, etc. [34, 35, 36]. Some variants associated with T2D, such as *HNF1A* p.I27L, *IDE3* p.T105A, *IRS1* p.S892G, and *INSR* p.A2G, although considered benign and determining quite conservative biochemical changes in the amino‐acid sequence, could influence the activity of the related proteins [37, 38, 39, 40, 41]. A similar effect can be expected by changes occurring at 5′‐UTR or intron variants that can impact transcription activity or alternative splicing. Most important, their cumulative effect is unpredictable and we cannot rule out the hypothesis that a “critical” cumulative, although benign, effect could contribute and prompt the malignancy. Interestingly, the genomic landscape of unselected metastatic CRC patients is rich in somatically mutated genes involved in T2D, as evidenced in previously published studies [42, 43, 44, 45, 46, 47, 48, 49]. The effects of these variants are unexplored, elusive, largely invaluable in functional and clinical point of views. In this light, lacking solid background data to prioritize these variants, we built a simple and intuitive score based on the sum of the number of genetic variants involved in both T2D and CRC (*CCND2, CDKN1B, CDKN2A, CDKN2B, CENPA, EML4, ID3, HNF1A, IGF1, IGF2, IGF1R, INSR, IRS1, IRS2,* and *TCF7L2*). Characterization of metastases from 11 not T2D pmCRC patients revealed that genes whose polymorphisms are generally recorded in T2D are also present in these not diabetic mCRC. By contrast, very few T2D‐associated polymorphisms were found in seven highly selected omCRC patients (not included in the analysis to avoid prognostic unbalances) having CRC as the only illness and who developed only one metastasis (4 pts with single nodule liver and 3 pts with single nodule lung metastasis) (“genuine” oligo‐metastatic single‐nodule patients) [50]. In detail, we observed that the diabetes‐associated *TCF7L2* variants were never present in omCRC patients. TCF7L2 is a pleomorphic transcription factor influencing several pathways involved in CRC and it acts as an effector of the Wnt pathway [8]. It confers the strongest association with T2D susceptibility and is located on chromosome 10q25.3, with rs7903146 being one of the most prevalent single nucleotide polymorphisms (SNPs) present in the *TCF7L2* gene [51]. The *TCF7L2* gene acts as a nuclear receptor in the Wnt signaling pathway that encodes a basic transcription factor 4 (TCF‐4) [52]. The β‐catenin is the most important element of the Wnt signaling pathway; when it binds to TCF‐4, the β‐catenin/TCF‐4 complex is formed; the complex is the final effector of the Wnt pathway that exerts a crucial role in the development of pancreatic islets and regulates hormone gene expression [53]. Interestingly, we found genetic variants of *TCF7L2* different from the more frequent c.450+33966C>T (corresponding to the rs7903146) [54]. This interesting result needs to be confirmed and explored in basic studies. We also found that T2D patients had both higher lymph nodes involvement at initial pathologic staging and a higher number of metastatic sites at diagnosis of advanced diseases. This biological CRC behavior in patients affected by T2D can be explained, at least in part, by the presence of a *TCF7L2* alteration. In fact, it was recently reported that *TCF7L2* plays an anti‐oncogenic role in CRC and is involved in the inhibition of motility and metastatization of CRC cells [55]. These effects can be at the bases of the frequent mutated status of *TCF7L2* in CRC and the association in our series between T2D and initial lymph node metastatization of the primary CRC. Another factor that can influence the metastatic potential of CRC in T2D patients is the increase of IGFs that is constantly observed in T2D and insulin resistance [7, 8, 56, 57, 58]. In fact, hyperstimulation of the IGFs/IGFR axis might also be involved in accounting for the lower rate of response observed in T2D patients, as already supposed by previous evidence [59]. In this regard, it is important to clarify that T2D patients of our series neither received a depotentiated/reduced chemotherapy nor experienced higher rates of toxicities.

Can the selected genes involved in CRC be considered as key driver genes? Surprisingly, excluding *TCF7L2* mutations implying loss of function, the selected genes are not directly involved in CRC carcinogenesis. In fact, *CDKN2B* and *HNF1A* deletions/inactivations have been evidenced in pancreatic adenocarcinoma [60, 61]. The *EML4‐ALK* “fusion‐type” oncogene is critical in driving oncogenesis in a subset of lung cancers [62]. Mutations in *ID3* are critical in Burkitt Lymphomas [63]. Interestingly, the IGF axis is a complex molecular network (including peptide‐ligands IGF1, IGF2, and insulin, and the receptors IGF1R, IGF2R, and INSR) that is involved in a wide variety of neoplasms such as prostate, breast, colorectal, and lung cancers [64]. However, it is frequently hyperactivated, probably as a consequence of upstream epi/genetic alterations. In fact, very recently HOX homeobox proteins (key oncogenic drivers particularly in hematopoietic malignancies) have been demonstrated to display their oncogenic potential by inducing the production of IGF1 [65].

Our study has some limitations. Most important, its intrinsic retrospective nature. This pitfall is partially limited by the mono‐institutional nature, which helps to reduce some biases linked to patients’ selection, treatments, and follow‐up. Furthermore, the mean glycemia value in our T2D patients at diagnosis was 152 mg·dL^−1^ (95% CI: 137–170 mg·dL^−1^); consequently, we could argue that diabetes was not well controlled in this T2D series. Analysis and discussion of the reason for hyperglycemia poor control is beyond the scope of this article; interestingly, a poor attitude to control T2D has been frequently observed after diagnosis of a cancer [66]. Therefore, we cannot exclude a direct effect of glucose on stimulating CRC and a worsening prognosis. This effect could also explain the reduced anticancer effect of metformin (used by all included patients) [67]. However, a better control of glycaemia could be associated with a better prognosis. Moreover, we have characterized the genetics of a limited number of patients of our series due to the high selection we made for a reliable and clean genotype/phenotype correlation. In this light, genetic results must be considered necessarily as having a hypothesis‐generating role. The number of T2D patients in our series was relatively small (41 patients); however, it is consistent with statistical expectations as well as with the incidence of the disease in the overall population (about 15%). Our series, in fact, was not enriched with T2D patients but it included the consecutive patients observed in the analyzed period.

## 5 Conclusion

We found that T2D is a negative prognostic factor for survival in CRC. The hypothesis that a “dosage effect” of gene variants not directly involved in determining cancer could concur with malignancy in CRC is fascinating and deserves to be studied in larger series. It could also provide a rational basis for innovative models of tumor progression and for integrated antitumor strategies.

## Conflict of interest

The authors declare no conflicts of interest.

### Author contributions

AO, MS, and MC (Michele Caraglia) conceptualized the study. AO, LC, and MC (Maurizio Capuozzo, affiliation no. 5) contributed to methodology. FP and MC contributed to design of software. AO, MC, and GN validated the data. AO, MC (Maurizio Capuozzo, affiliation no. 3), VG (Valerio Gigantino), VG (Vincenza Granata) made formal analysis. LC, VG (Valerio Gigantino), GS, DF, SZ, and AL investigated the study. MC (Michele Caraglia) contributed to resources. AO, MS, and MC (Michele Caraglia) contributed to data curation. AO, MS, and MC (Michele Caraglia), GN contributed to writing/original draft preparation. GN, FP, and MC (Marco Cascella) contributed to writing/review and editing. MC (Michele Caraglia) supervised the study.

## Supporting information


**Fig. S1.** List of genes characterized through TrusightOncology 500.

**Fig. S2.** Phenolyzer network visualization legend.

**Table S1.** Connections between genes involved in colorectal cancer and T2D (Type 2 diabetes) analyzed by TrisghtOncology 500.

**Table S2.** Incidence of G3/G4 adverse event *per* patient in first‐ and second‐line chemotherapies according to presence or not of T2D (Type 2 diabetes).

**Table S3.** Incidence of reasons for chemotherapy dose reductions in first‐ and second‐line chemotherapies according to presence or not of T2D (Type 2 diabetes).

## Data Availability

The data that support the findings of this study are available on request from Dr. Alessandro Ottaiano (a.ottaiano@istitutotumori.na.it). The data are not publicly available due to privacy or ethical restrictions.
