# Supplementary material for: Metastatic colorectal cancer and type 2 diabetes: prognostic and genetic interactions
Source: Mol Oncol. 2021 Nov 19;16(2):319–32. doi: 10.1002/1878-0261.13122 (PMC8763648; doi:10.1002/1878-0261.13122)
Supplement: Supplementary file 1 — Fig. S1. List of genes characterized through TrusightOncology 500. [file MOL2-16-319-s003.pptx]

## Slide 1
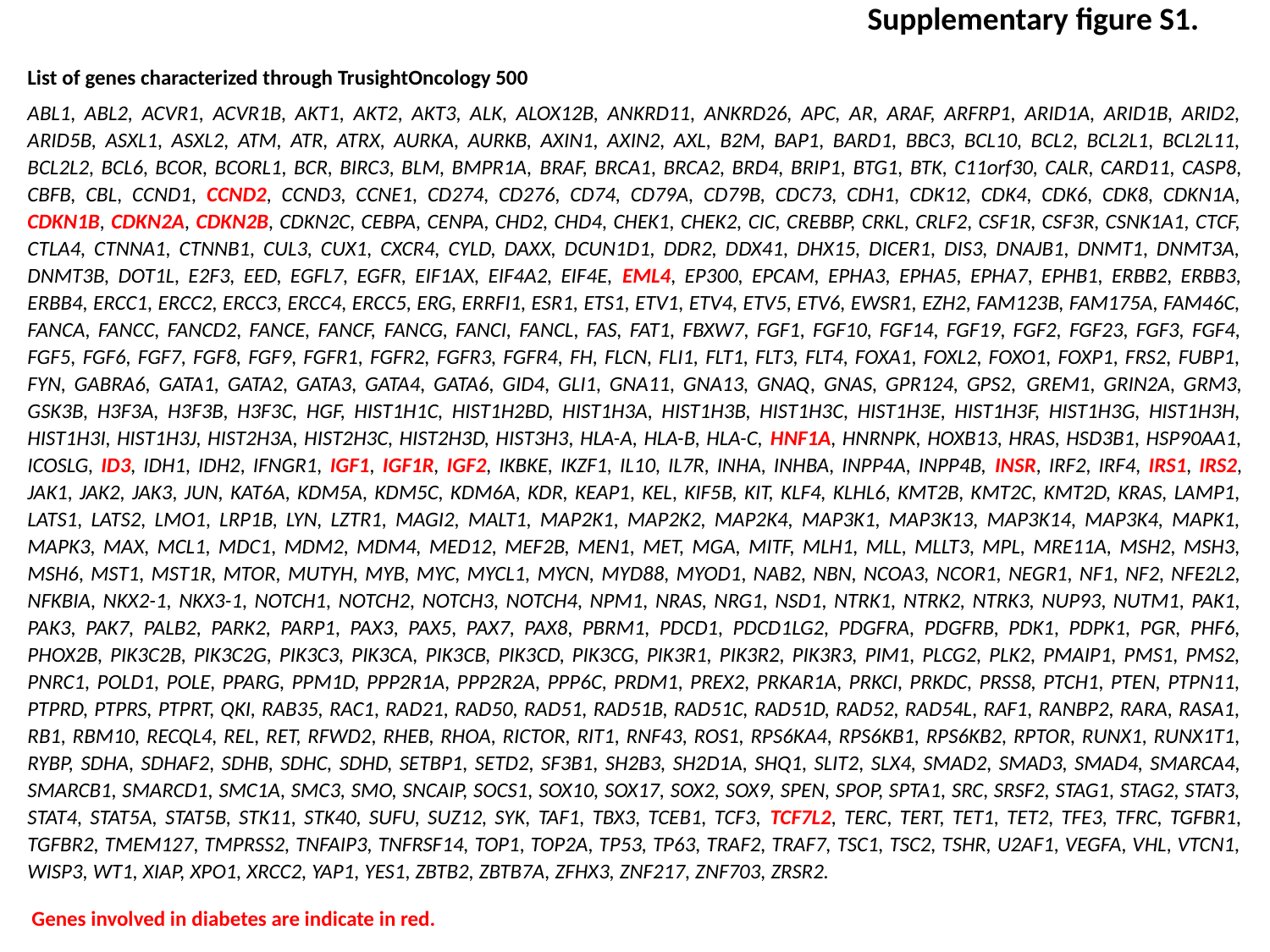

Supplementary figure S1.
List of genes characterized through TrusightOncology 500
ABL1, ABL2, ACVR1, ACVR1B, AKT1, AKT2, AKT3, ALK, ALOX12B, ANKRD11, ANKRD26, APC, AR, ARAF, ARFRP1, ARID1A, ARID1B, ARID2, ARID5B, ASXL1, ASXL2, ATM, ATR, ATRX, AURKA, AURKB, AXIN1, AXIN2, AXL, B2M, BAP1, BARD1, BBC3, BCL10, BCL2, BCL2L1, BCL2L11, BCL2L2, BCL6, BCOR, BCORL1, BCR, BIRC3, BLM, BMPR1A, BRAF, BRCA1, BRCA2, BRD4, BRIP1, BTG1, BTK, C11orf30, CALR, CARD11, CASP8, CBFB, CBL, CCND1, CCND2, CCND3, CCNE1, CD274, CD276, CD74, CD79A, CD79B, CDC73, CDH1, CDK12, CDK4, CDK6, CDK8, CDKN1A, CDKN1B, CDKN2A, CDKN2B, CDKN2C, CEBPA, CENPA, CHD2, CHD4, CHEK1, CHEK2, CIC, CREBBP, CRKL, CRLF2, CSF1R, CSF3R, CSNK1A1, CTCF, CTLA4, CTNNA1, CTNNB1, CUL3, CUX1, CXCR4, CYLD, DAXX, DCUN1D1, DDR2, DDX41, DHX15, DICER1, DIS3, DNAJB1, DNMT1, DNMT3A, DNMT3B, DOT1L, E2F3, EED, EGFL7, EGFR, EIF1AX, EIF4A2, EIF4E, EML4, EP300, EPCAM, EPHA3, EPHA5, EPHA7, EPHB1, ERBB2, ERBB3, ERBB4, ERCC1, ERCC2, ERCC3, ERCC4, ERCC5, ERG, ERRFI1, ESR1, ETS1, ETV1, ETV4, ETV5, ETV6, EWSR1, EZH2, FAM123B, FAM175A, FAM46C, FANCA, FANCC, FANCD2, FANCE, FANCF, FANCG, FANCI, FANCL, FAS, FAT1, FBXW7, FGF1, FGF10, FGF14, FGF19, FGF2, FGF23, FGF3, FGF4, FGF5, FGF6, FGF7, FGF8, FGF9, FGFR1, FGFR2, FGFR3, FGFR4, FH, FLCN, FLI1, FLT1, FLT3, FLT4, FOXA1, FOXL2, FOXO1, FOXP1, FRS2, FUBP1, FYN, GABRA6, GATA1, GATA2, GATA3, GATA4, GATA6, GID4, GLI1, GNA11, GNA13, GNAQ, GNAS, GPR124, GPS2, GREM1, GRIN2A, GRM3, GSK3B, H3F3A, H3F3B, H3F3C, HGF, HIST1H1C, HIST1H2BD, HIST1H3A, HIST1H3B, HIST1H3C, HIST1H3E, HIST1H3F, HIST1H3G, HIST1H3H, HIST1H3I, HIST1H3J, HIST2H3A, HIST2H3C, HIST2H3D, HIST3H3, HLA-A, HLA-B, HLA-C, HNF1A, HNRNPK, HOXB13, HRAS, HSD3B1, HSP90AA1, ICOSLG, ID3, IDH1, IDH2, IFNGR1, IGF1, IGF1R, IGF2, IKBKE, IKZF1, IL10, IL7R, INHA, INHBA, INPP4A, INPP4B, INSR, IRF2, IRF4, IRS1, IRS2, JAK1, JAK2, JAK3, JUN, KAT6A, KDM5A, KDM5C, KDM6A, KDR, KEAP1, KEL, KIF5B, KIT, KLF4, KLHL6, KMT2B, KMT2C, KMT2D, KRAS, LAMP1, LATS1, LATS2, LMO1, LRP1B, LYN, LZTR1, MAGI2, MALT1, MAP2K1, MAP2K2, MAP2K4, MAP3K1, MAP3K13, MAP3K14, MAP3K4, MAPK1, MAPK3, MAX, MCL1, MDC1, MDM2, MDM4, MED12, MEF2B, MEN1, MET, MGA, MITF, MLH1, MLL, MLLT3, MPL, MRE11A, MSH2, MSH3, MSH6, MST1, MST1R, MTOR, MUTYH, MYB, MYC, MYCL1, MYCN, MYD88, MYOD1, NAB2, NBN, NCOA3, NCOR1, NEGR1, NF1, NF2, NFE2L2, NFKBIA, NKX2-1, NKX3-1, NOTCH1, NOTCH2, NOTCH3, NOTCH4, NPM1, NRAS, NRG1, NSD1, NTRK1, NTRK2, NTRK3, NUP93, NUTM1, PAK1, PAK3, PAK7, PALB2, PARK2, PARP1, PAX3, PAX5, PAX7, PAX8, PBRM1, PDCD1, PDCD1LG2, PDGFRA, PDGFRB, PDK1, PDPK1, PGR, PHF6, PHOX2B, PIK3C2B, PIK3C2G, PIK3C3, PIK3CA, PIK3CB, PIK3CD, PIK3CG, PIK3R1, PIK3R2, PIK3R3, PIM1, PLCG2, PLK2, PMAIP1, PMS1, PMS2, PNRC1, POLD1, POLE, PPARG, PPM1D, PPP2R1A, PPP2R2A, PPP6C, PRDM1, PREX2, PRKAR1A, PRKCI, PRKDC, PRSS8, PTCH1, PTEN, PTPN11, PTPRD, PTPRS, PTPRT, QKI, RAB35, RAC1, RAD21, RAD50, RAD51, RAD51B, RAD51C, RAD51D, RAD52, RAD54L, RAF1, RANBP2, RARA, RASA1, RB1, RBM10, RECQL4, REL, RET, RFWD2, RHEB, RHOA, RICTOR, RIT1, RNF43, ROS1, RPS6KA4, RPS6KB1, RPS6KB2, RPTOR, RUNX1, RUNX1T1, RYBP, SDHA, SDHAF2, SDHB, SDHC, SDHD, SETBP1, SETD2, SF3B1, SH2B3, SH2D1A, SHQ1, SLIT2, SLX4, SMAD2, SMAD3, SMAD4, SMARCA4, SMARCB1, SMARCD1, SMC1A, SMC3, SMO, SNCAIP, SOCS1, SOX10, SOX17, SOX2, SOX9, SPEN, SPOP, SPTA1, SRC, SRSF2, STAG1, STAG2, STAT3, STAT4, STAT5A, STAT5B, STK11, STK40, SUFU, SUZ12, SYK, TAF1, TBX3, TCEB1, TCF3, TCF7L2, TERC, TERT, TET1, TET2, TFE3, TFRC, TGFBR1, TGFBR2, TMEM127, TMPRSS2, TNFAIP3, TNFRSF14, TOP1, TOP2A, TP53, TP63, TRAF2, TRAF7, TSC1, TSC2, TSHR, U2AF1, VEGFA, VHL, VTCN1, WISP3, WT1, XIAP, XPO1, XRCC2, YAP1, YES1, ZBTB2, ZBTB7A, ZFHX3, ZNF217, ZNF703, ZRSR2.
Genes involved in diabetes are indicate in red.
