# Supplementary figures and images for: Metastatic colorectal cancer and type 2 diabetes: prognostic and genetic interactions
Source: Mol Oncol. 2021 Nov 19;16(2):319–32. doi: 10.1002/1878-0261.13122 (PMC8763648; doi:10.1002/1878-0261.13122)

## Slide 1
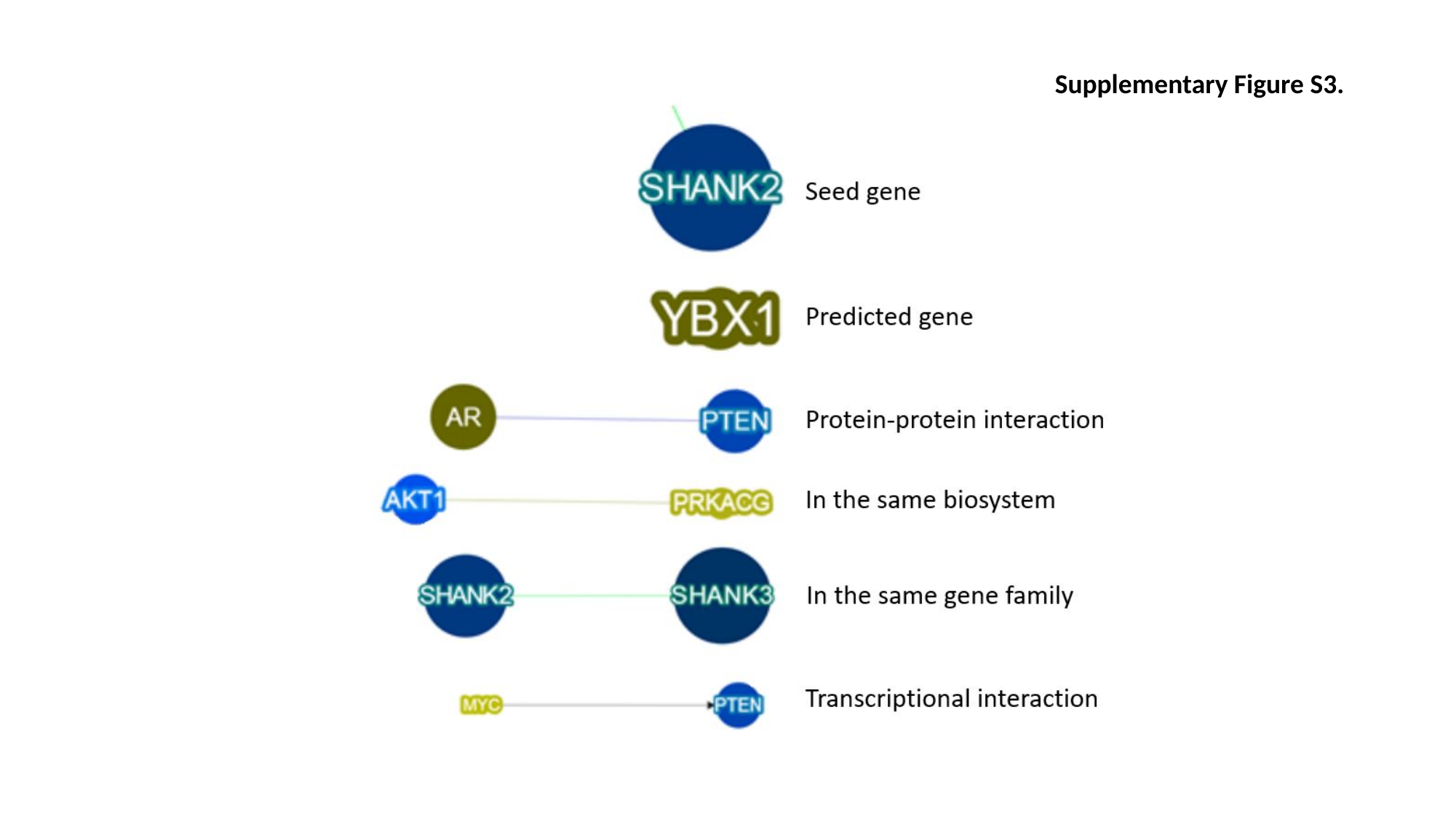

Supplementary Figure S3.

Supplement: Supplementary file 2 — Fig. S2. Phenolyzer network visualization legend. [file MOL2-16-319-s001.pptx]
