# Supplementary material for: Metastatic colorectal cancer and type 2 diabetes: prognostic and genetic interactions
Source: Mol Oncol. 2021 Nov 19;16(2):319–32. doi: 10.1002/1878-0261.13122 (PMC8763648; doi:10.1002/1878-0261.13122)
Supplement: Supplementary file 3 — Table S1. Connections between genes involved in colorectal cancer and T2D (Type 2 diabetes) analyzed by TrisghtOncology 500. [file MOL2-16-319-s002.doc]

**Supplementary Table S1.** Connections between genes involved in CRC and T2D analyzed by TrisghtOncology^TM^ 500.

| **Gene** | **Description** | **Physiologic function** | **Chromosome localization** | **Role in cancer** | **Role in type 2 diabetes** |
| --- | --- | --- | --- | --- | --- |
| **CCND2** | Cyclin D2. | Regulation of cyclin-dependent kinase. | 12 | Cell growth and invasiveness. | Molecular link between glucose levels and β-cell replication. |
| **CDKN1B** | Cyclin-dependent kinase inhibitor 1B. | Regulation of cyclin-dependent kinase. | 12 | Cell growth and invasiveness. | Regulation of β-cell mass. |
| **CDKN2A** | Cyclin-dependent kinase inhibitor 2A. | Regulation of cyclin-dependent kinase. | 9 | Cell growth and invasiveness. | Regulation of β-cell insulin secretion. |
| **CDKN2B** | Cyclin-dependent kinase inhibitor 2B. | Regulation of cyclin-dependent kinase. | 9 | Cell growth and invasiveness. | Regulation of β-cell insulin secretion. |
| **CENPA** | Centromeric protein A. | It is a variant of**histone H3 and a critical factor influencing the kinetochore positions.** | 2 | Cancer initiation and progression. | Involved in insulin signaling. |
| **EML4** | Echinoderm microtubule associated protein like 4. | Protein required for the organization of the mitotic spindle and attachment of kinetochores to microtubules. | 2 | Cell growth and differentiation. | Significantly associated with type II diabetes but function largely unknown. |
| **ID3** | Inhibitor of DNA-binding protein-3. | Regulation of transcription. | 1 | Cell growth and differentiation. | Involved in insulin resistance and atherogenesis. |
| **HNF1A** | Hepatocyte nuclear factor-1 alpha. | Transcription factor involved in regulating genes with pleiotropic actions. | 12 | Cell growth and differentiation. | Regulation of hepatic and pancreatic genes to maintain glucose homeostasis. |
| **IGF1** | Insulin-like growth factor 1. | It is a primary mediator of the effects of [growth hormone](https://en.wikipedia.org/wiki/Growth_hormone) (GH). | 12 | Cell growth, differentiation and invasiveness. | Insulin signaling. |
| **IGF2** | Insulin-like growth factor 2. | The major role of IGF2 is cell growth stimulation during [gestation](https://en.wikipedia.org/wiki/Gestation). | 11 | Cell growth, differentiation and invasiveness. | Insulin signaling. |
| **IGF1R** | Insulin growth factor-1 receptor. | It mediates actions of insulin-like growth factor 1. | 15 | Cell growth, differentiation and invasiveness. | Insulin signaling. |
| **INSR** | Insulin receptor. | Transmembrane tyrosine kinase receptor activated by insulin, IGF1, and IGF2. | 19 | Cell growth, differentiation and invasiveness. | Insulin signaling. |
| **IRS1** | Insulin receptor substrate 1. | It is a signaling adapter protein from the insulin and IGF1. | 2 | Cancer initiation and progression. | Insulin and IGF1 signaling. |
| **IRS2** | Insulin receptor substrate 2. | It is a signaling adapter protein from the insulin, IGF1 and cytokines (including IL-4)-mediated signals. | 13 | Cancer initiation and progression. | Insulin, IGF1 and cytokines signaling. |
| **TCF7L2** | Transcription factor 7-like 2. | Transcription factor influencing several biological pathways, including the [Wnt signaling pathway](https://en.wikipedia.org/wiki/Wnt_signaling_pathway). | 10 | Cell growth and invasiveness. | TCF7L2 polymorphisms can increase susceptibility to type 2 diabetes by decreasing the production of glucagon-like peptide-1 (GLP-1). |
