# Supplementary material for: Metastatic colorectal cancer and type 2 diabetes: prognostic and genetic interactions
Source: Mol Oncol. 2021 Nov 19;16(2):319–32. doi: 10.1002/1878-0261.13122 (PMC8763648; doi:10.1002/1878-0261.13122)
Supplement: Supplementary file 4 — Table S2. Incidence of G3/G4 adverse event per patient in first‐ and second‐line chemotherapies according to presence or not of T2D (Type 2 diabetes). [file MOL2-16-319-s005.docx]

**Supplementary Table S2.** Incidence of G3/G4 adverse event *per* patient in first- and second-line chemotherapies according to presence or not of T2D (Type 2 diabetes).

| **Toxicity** | **T2D** | | ****P*** |
| --- | --- | --- | --- |
|  | **No** | **Yes** |  |
| **Hematologic** |  |  |  |
| Neutropenia | 10 | 3 |  |
| Anemia | 3 | 0 |  |
| Thrombocytopenia | 2 | 1 |  |
| Total | 15 | 4 |  |
| **Non-hematologic** |  |  |  |
| Asthenia | 7 | 2 |  |
| Diarrhea | 4 | 1 |  |
| Nausea | 2 | 0 |  |
| Mucositis | 1 | 0 |  |
| Cardiovascular | 0 | 1 |  |
| AST/ALT increase | 1 | 0 |  |
| Epistaxis | 1 | 0 |  |
| Blood bilirubin increase | 0 | 1 |  |
| Kidney injury | 0 | 1 |  |
| Allergic | 1 | 0 |  |
| Total | 17 | 6 | *P*=0.7064 |

ALT: Alanine transaminase; AST: Aspartate transaminase. **P* at chi-square test.
