# Supplementary material for: Metastatic colorectal cancer and type 2 diabetes: prognostic and genetic interactions
Source: Mol Oncol. 2021 Nov 19;16(2):319–32. doi: 10.1002/1878-0261.13122 (PMC8763648; doi:10.1002/1878-0261.13122)
Supplement: Supplementary file 5 — Table S3. Incidence of reasons for chemotherapy dose reductions in first‐ and second‐line chemotherapies according to presence or not of T2D (Type 2 diabetes). [file MOL2-16-319-s004.docx]

**Supplementary Table S3.** Incidence of reasons for chemotherapy dose reductions in first- and second-line chemotherapies according to presence or not of T2D (Type 2 diabetes).

|  | **T2D** | | ****P*** |
| --- | --- | --- | --- |
|  | **No** | **Yes** |  |
| **Reasons for dose treatment reduction** |  |  |  |
| Hematologic toxicity | 32 | 8 |  |
| Non-hematologic toxicity | 18 | 5 |  |
| Patient request | 5 | 0 |  |
| No dose reduction | 107 | 28 | *P*=0.7232 |

**P* at chi-square test.
